# Trace conditioning in insects—keep the trace!

**DOI:** 10.3389/fphys.2013.00067

**Published:** 2013-08-23

**Authors:** Kristina V. Dylla, Dana S. Galili, Paul Szyszka, Alja Lüdke

**Affiliations:** ^1^Department of Biology, Neurobiology, University of KonstanzKonstanz, Germany; ^2^Behavioral Genetics, Max-Planck Institute for NeurobiologyMartinsried, Germany

**Keywords:** trace conditioning, insects, stimulus trace, learning, olfaction

## Abstract

Trace conditioning is a form of associative learning that can be induced by presenting a conditioned stimulus (CS) and an unconditioned stimulus (US) following each other, but separated by a temporal gap. This gap distinguishes trace conditioning from classical delay conditioning, where the CS and US overlap. To bridge the temporal gap between both stimuli and to form an association between CS and US in trace conditioning, the brain must keep a neural representation of the CS after its termination—a stimulus trace. Behavioral and physiological studies on trace and delay conditioning revealed similarities between the two forms of learning, like similar memory decay and similar odor identity perception in invertebrates. On the other hand differences were reported also, like the requirement of distinct brain structures in vertebrates or disparities in molecular mechanisms in both vertebrates and invertebrates. For example, in commonly used vertebrate conditioning paradigms the hippocampus is necessary for trace but not for delay conditioning, and *Drosophila* delay conditioning requires the Rutabaga adenylyl cyclase (Rut-AC), which is dispensable in trace conditioning. It is still unknown how the brain encodes CS traces and how they are associated with a US in trace conditioning. Insects serve as powerful models to address the mechanisms underlying trace conditioning, due to their simple brain anatomy, behavioral accessibility and established methods of genetic interference. In this review we summarize the recent progress in insect trace conditioning on the behavioral and physiological level and emphasize similarities and differences compared to delay conditioning. Moreover, we examine proposed molecular and computational models and reassess different experimental approaches used for trace conditioning.

## Introduction

Actions may have delayed, rather than immediate consequences. If you have ever woken up with a terrible headache the morning after drinking too much tequila, you probably had a feeling of nausea the next time you encountered the taste and smell of tequila and may have refrained from drinking it. Although there was a temporal dissociation between the two events—the stimulus and the negative consequence—an aversive association was formed.

**Figure 1:**
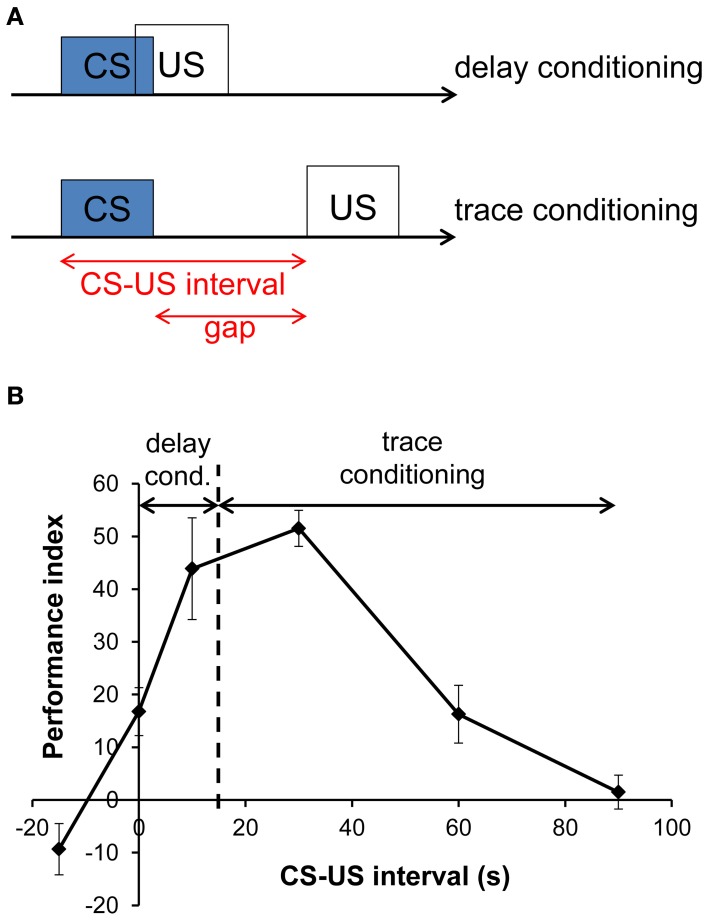
**Experimental design and memory performance in delay and trace conditioning. (A)** Experimental design of delay and trace conditioning. In delay conditioning, the conditioned stimulus (CS) and the unconditioned stimulus (US) overlap, whereas they are separated by a gap in trace conditioning. The time between the onset of the CS and the onset of the US is termed CS–US interval. **(B)** Memory performance after aversive olfactory conditioning in *Drosophila melanogaster* as a function of CS–US interval. Positive and negative scores indicate conditioned avoidance and conditioned approach, respectively. A 15 s long odor presentation serves as CS. Its end is indicated by a dashed line. The US consists of four electric shocks at 90 V applied within 16 s. Trials with a CS–US interval ≤15 s are termed delay conditioning and those with a CS–US interval >15 s are termed trace conditioning. Adapted from Tanimoto et al. (2004) with changes and with permission from the author.

To account for the possibility to associate stimuli which are separated in time, the preceding conditioned stimulus (CS) must induce a representation in the form of a stimulus trace in the neuronal network, which persists for a time after the stimulus has terminated. The stimulus trace can then be associated with the following reinforcing unconditioned stimulus (US). This form of learning where CS and US are separated by a temporal gap is termed trace conditioning, in contrast to delay conditioning, where both CS and US occur with a temporal overlap (Figure 1A). We refer to the CS–US interval as the time span between CS onset and US onset, while the time span between the CS offset and US onset is termed gap (Figure 1A).

The capability to associate two temporally separated stimuli is vital for animals, since in nature the cause and effect of a behavior are often not contiguous. Additionally, the perceived features of objects are constantly changing with time and location. Animals must memorize and integrate these changing features for object identification and tracking. For example, when a bee approaches a flower, the flower's shape and color may change drastically in the bee's perception. Still, the bee should learn to associate the initial visual stimulus with the food reward in order to initiate the approach next time (Opfinger, 1931; Menzel, 1968; Grossmann, 1970).

In 1927, Ivan Pavlov had already noted that dogs responded with increased saliva production to the CS alone after training with a whistle-sound CS and a food reward US, which were separated by several minutes (Pavlov, 1927). In this case, the salivary response was delayed proportional to the interval between the CS and the US, suggesting that the dogs learned to anticipate the US.

Since then, trace conditioning was shown in a variety of paradigms including eyeblink conditioning (Smith et al., 1969), fear conditioning (King, 1965), autoshaping (Gibbon et al., 1977), and conditioned taste aversion (Barker and Smith, 1974) using different CS (auditory, visual, gustatory) and US (food, water, shock, LiCl) in organisms including dogs, rats, pigeons, rabbits, and humans (reviewed in Rescorla, 1988).

Are the memories formed during delay and trace conditioning governed by different neuronal pathways? In mammals, for example, the hippocampus is additionally required for trace but not for delay eyeblink conditioning (Solomon et al., 1986; Woodruff-Pak and Disterhoft, 2008) and the state of awareness can play a role for trace conditioning in humans (Clark and Squire, 1998; Clark et al., 2002; Christian and Thompson, 2003). Therefore, trace memory is proposed to be qualitatively distinct from delay memory. Although commonly accepted, this view has been challenged (LaBar and Disterhoft, 1998). The demands on neural resources increase with task complexity for both trace and delay conditioning (Knuttinen et al., 2001; Carter et al., 2003). Thus the differential requirement of the hippocampus for trace conditioning might be a result of task complexity and not of the discontinuity between stimulus and reinforcement (Carrillo et al., 2000; Beylin et al., 2001; Walker and Steinmetz, 2008; Kehoe et al., 2009). The necessity of awareness in trace conditioning was also challenged in recent studies which demonstrated that humans can also learn trace conditioning without awareness, such as when asleep (Arzi et al., 2012) or in a vegetative state (Bekinschtein et al., 2009). How delay and trace conditioning differ in their anatomical and physiological basis is still an open question? Where and how is the stimulus trace maintained until US arrival? Is the trace actively being kept in the brain or is it a passive decay of activity originated from the stimulus? What are the molecular correlates of the stimulus trace?

In recent years several approaches to study the cellular and molecular mechanisms of stimulus traces and trace memories have been performed in insects (Ito et al., 2008; Tomchik and Davis, 2009; Galili et al., 2011; Shuai et al., 2011; Szyszka et al., 2011), taking advantage of their ability to solve the trace conditioning task generally faster than vertebrates. Another beneficial aspect of using insects is based on their simpler brains which allow easier access for physiological measures and enable genetic and molecular manipulations. In this review, we summarize the findings regarding the behavioral, molecular, anatomical, and modeling data on trace conditioning in insects and point out the knowledge gaps that still wait to be filled.

## Special and common features of trace conditioning

Insect trace conditioning has been mainly conducted in three behavioral paradigms. Early experiments were performed with freely flying honeybees, where the animals learned to associate a color which was presented during the approach of a food source, but not during feeding with a food reward. Successful conditioning was observed as a preference for the trained color during future landings (Opfinger, 1931; Menzel, 1968; Grossmann, 1970). Later studies were done in harnessed animals, pairing odor stimuli with a temporally separated sugar reward (*Apis mellifera*: Menzel, 1983; Szyszka et al., 2011; *Manduca sexta*: Ito et al., 2008). Associative memory formation was measured as proboscis extension reflex (PER) in response to the odor presented alone. In *Drosophila melanogaster*, aversive olfactory conditioning in the T-maze is the most commonly used paradigm for trace conditioning (Tully and Quinn, 1985; Tanimoto et al., 2004; Galili et al., 2011; Shuai et al., 2011). Here, a group of animals is trained to associate an odor with the following electric shock. During testing, animals have to choose between the previously punished odor and either a different odor or pure air.

The paradigm diversity may account for observed differences in learning performance when comparing trace with delay conditioning, within and between species. *Drosophila*, for example, is able to reach the same learning asymptote in olfactory delay and trace conditioning in the T-maze (Galili et al., 2011). In contrast, honeybees (*Apis mellifera*) did not reach the same learning asymptote in trace conditioning compared to delay conditioning, as measured with PER (up to 19 training trials in Menzel et al., 1993; up to six training trials in Szyszka et al., 2011). Further experiments are required to clarify if these disparities are paradigm-dependent effects.

In addition to these differences, shared features of delay and trace conditioning were also revealed, such as similar perception of odor identity and similar memory decay curves (Galili et al., 2011; Szyszka et al., 2011). Also the shape of CS–US interval functions showed resemblance across species and paradigms: Figure 1B shows a CS–US interval function in insects, which is strikingly similar to that of mammals (Rescorla, 1988); likewise, the obtained curve for insect visual learning is similar to that for olfactory learning (Menzel, 1983).

One remarkable phenomenon in trace conditioning is the existence of paradigm learning. Previous trace conditioning improved pigeons' learning performance in subsequent trace conditioning with longer CS–US intervals (Lucas et al., 1981). The same effect was observable in insects. One-trial trace conditioning with a short CS–US interval enabled honeybees to succeed in initially unsolvable 1-trial trace conditioning with an extended CS–US interval (Szyszka et al., 2011). Similarly, *Drosophila* trained in five trials with increasing CS–US interval learned better than flies presented with the reverse order of intervals (Galili et al., 2011).

These studies show, that animals gain experience in trace conditioning which facilitates learning during subsequent trace conditioning. But what kind of experience is this? In honeybees, response latency during the test was shorter following training with delay conditioning (simultaneous CS and US onset) than with trace conditioning. Even if response latency did not correlate with the CS–US interval during trace conditioning (Szyszka et al., 2011), this result suggests that during training with trace conditioning animals learn something about US timing. This is in accordance with Pavlov (1927) who found a later conditioned response in dogs after trace conditioning.

What happens in the brain while the animal is waiting for the reinforcement? At the level of neuronal correlates, there are two possible ways how the previous successful performance in trace conditioning tasks can help bridging the longer gap in following trials. The first mechanism is prolongation of the CS trace until the arrival of the US, altering the CS representation pathway. Another possible mechanism is the activation of US-representing neurons during the CS (US anticipation), assuming that the animals learn a causal connection between the separated CS and US [as is the case in monkeys, reviewed by Schultz (2006)]. Since a single training trial is enough for some insects to learn, US anticipation can be excluded as a possible explanation for 1-trial trace learning. Nonetheless, after several trials, US anticipation may develop. The proposed mechanisms may also act together to improve consecutive trace conditioning trials. Behaviorally, US anticipation can enhance the CS saliency, so that the animal will assign greater importance to the fading CS trace by during consecutive trials.

An interesting characteristic of trace conditioning in vertebrates is that filling the gap with another stimulus enhances learning (Kamin, 1965; Kaplan, 1984), whereas interference is detrimental to non-associative short-term memories like habituation and sensitization.

This learning enhancement was also shown in olfactory trace conditioning in honeybees, where a second CS within the gap strengthened the association between the first CS and the US (Szyszka et al., 2011). How does an additional stimulus during the gap improve trace conditioning? It might act as a distinguishing feature from the background, which changes the environmental context, creating a “bridge” between CS and US. In a natural environment where stimuli follow each other with varying time intervals, such a distinguishing feature may help the animal to resolve the temporal ambiguity between the CS and US, i.e., whether a US is related to a preceding CS, or to a following CS (Beylin et al., 2001).

## When is the odor trace initiated?

Depending on the stimulus length, both the onset and the offset of a stimulus can serve as a CS (Kehoe et al., 2009). This was found in rabbit nictitating membrane trace conditioning (where a tone is associated with an air puff to the eye) when a CS of several hundred milliseconds or longer was used (Desmond and Moore, 1991; Kehoe and Weidemann, 1999; Kehoe and Macrae, 2002).

Behavioral studies in honeybees (Szyszka et al., 2011) and *Manduca sexta* (Ito et al., 2008) indicated that the initial part of an odor stimulus and not the late phase or odor offset triggers the stimulus trace, whereas in *Drosophila* the odor offset seems to elicit a trace (Galili et al., 2011). The different time points of trace initiation might explain why the CS–US interval learned by *Drosophila* (CS–US interval: 25 s; gap: 15 s; Galili et al., 2011) was longer than that learned by honeybees (CS–US interval: 6 s; gap: 5.5 s; Szyszka et al., 2011). The observed behavioral differences might indeed be a matter of stimulus length (Kehoe et al., 2009), which varied between 0.5 s in the honeybee study to 10 s in the *Drosophila* study.

Ecologically, the observed differences in trace initiation might account for species-specific requirements. Fast flying insects such as honeybees and moths possess a remarkable ability to identify and track a single odor in a highly turbulent, multi-odor background. This ability relies on analyzing and remembering the temporal structure of odor plumes, which contain information about the distance and location of the odor source (Vickers, 2000; Cardé and Willis, 2008). Therefore, fast flying insects may need to stay receptive for new odors which they might encounter during flight. They may need to remember when they encounter an odor plume rather than when they leave it. In contrast, slow flying insects, such as *Drosophila*, live in a more static olfactory environment. Living in such a habitat, it might be more important to be sensitive to concentration gradients rather than to on- and offsets of fast fluctuating odors. Altogether, species-specific differences have to be considered when searching for the neural correlates of CS traces and CS–US association during trace conditioning.

Finally, different methods of odor delivery in the behavioral studies [automatic in Szyszka et al. (2011) vs. manual in Galili et al. (2011)] may account for the observed differences in trace initiation between honeybees and *Drosophila*. These behavioral studies indicated the time windows in which the stimulus traces in different species are triggered. This information is crucial to focus on the particular time window in physiological experiments aiming at the identification of stimulus traces.

## No evidence yet for trace-related neural activity

**Figure 2:**
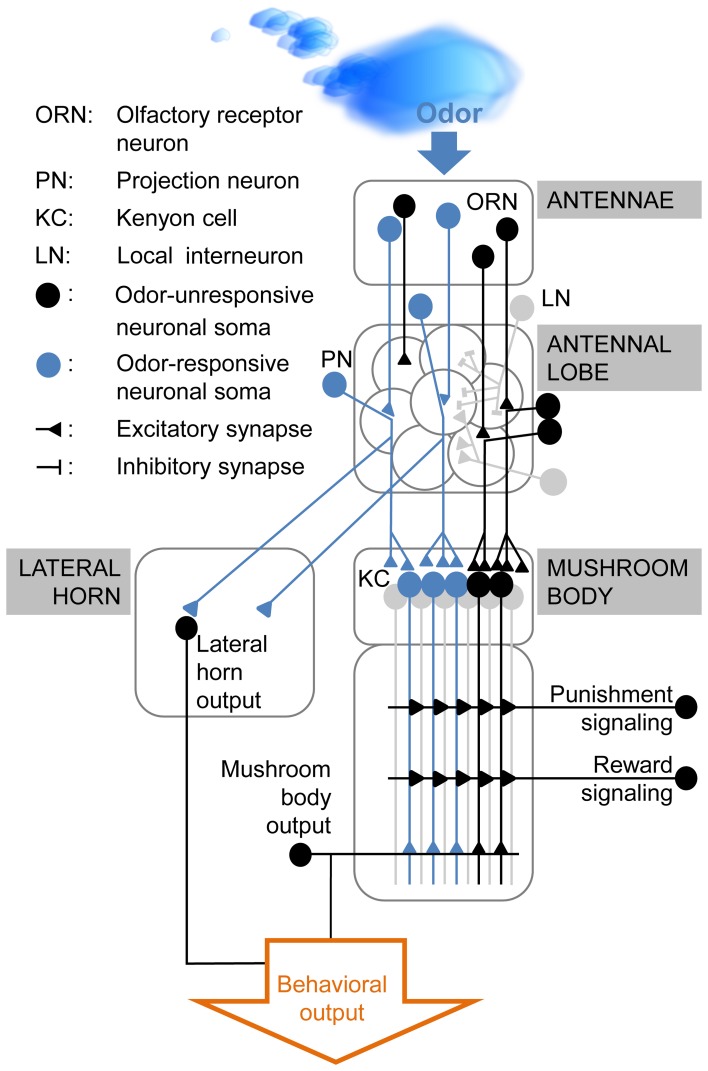
**Simplified diagram of olfactory processing in *Drosophila melanogaster*.** Odors activate distinct sets of olfactory receptor neurons (ORNs) in the fly's antenna. ORNs which express the same receptor converge onto the same glomerulus in the antennal lobe. Thus, each odor induces a unique glomerular activity pattern. This pattern is modulated by the interaction between ORNs, excitatory and inhibitory local interneurons (LNs) and projection neurons (PNs). The PNs relay the odor information to the lateral horn (which is assumed to mediate the innate odor response) and to the mushroom body intrinsic Kenyon cells (KCs). Upon reinforcement, KCs receive input from modulatory neurons, signaling either punishment or reward. Coincident activation of these neurons and the odor-responsive KCs in delay conditioning is believed to modify the output in these KCs. This modification changes the fly's behavior to the previously reinforced odor stimulus. For trace conditioning the underlying mechanism is yet unknown, but it seems likely that the necessary modifications occur in the olfactory pathway and/or pathway-associated neurons.

Where is the information about the CS stored until the US arrives? The insect olfactory pathway (Figure 2) starts at the antennae where odors activate odor-specific subsets of olfactory receptor neurons (ORNs). ORNs transmit odor information to the antennal lobe, the primary brain area for olfactory processing. Here, ORN axons interact with excitatory and inhibitory local interneurons (LNs) and with projection neurons (PNs) which conduct the information to higher order neurons, like the Kenyon cells (KCs) in the mushroom body and lateral horn neurons (Figure 2). The olfactory trace might be located in any of these neuron types or in other neurons outside the olfactory pathway.

There is good evidence that odor identity is encoded in antennal lobe odor response patterns. The perceived odor similarity (extracted from behavioral odor generalization experiments) corresponds to the physiological odor similarity (as measured with calcium imaging, comparing odor-evoked combinatorial glomerular activity patterns) during odor presentation. This was shown in the honeybee (Guerrieri et al., 2005) and *Drosophila* (Niewalda et al., 2011).

If ORNs or PNs encode the trace, their physiological post-odor similarity profile should follow the same correlation as the behavioral similarity profile and predict the perceived similarity profile during trace conditioning. However, such correlation between physiological post-odor activity and behavioral generalization after trace conditioning was neither found in *Drosophila* ORNs (Galili et al., 2011) nor in honeybee PNs (Szyszka et al., 2011). These findings indicate that the odor trace should be located downstream of the PNs. Consistently, analyzing action potential firing patterns in honeybee PNs, Nawrot (2012) found that the correlation between the initial and later phases of odor response patterns was high, but rapidly decreased with odor offset (Krofczik et al., 2008). Similarly, post-odor activity in mouse mitral cells is odor specific but different from the odor response (Bathellier et al., 2008). The common finding of specific post-odor activity in different cell populations in the olfactory pathway suggests that this feature, though it does not correlate directly with trace conditioning, may be an evolutionarily conserved property. It remains to be shown whether such post-odor response patterns have functional relevance or whether they are a mere byproduct of odor processing.

From these findings it can be concluded that an olfactory stimulus trace does not consist of persistent neuronal activity in ORNs or PNs. But these findings do not rule out the possibility that the stimulus trace is encoded in antennal lobe LNs, or in any of these neuron types as biochemical modifications (Perisse and Waddell, 2011). Subtle network activity, such as changes in the correlation of glomerular spontaneous activity after the presentation of an odor (Galan et al., 2006) might also be an underlying mechanism.

A more promising brain structure, however, is the mushroom body which is the site where different stimulus modalities converge and associative olfactory learning occurs (Erber et al., 1980; Heisenberg et al., 1985; Menzel, 2001). Consistently, Shuai et al. (2011) suggested a role of *Drosophila* KCs in trace conditioning, based on studies with Rac which is a small G protein belonging to the Rho family of GTPases. Elevated Rac activity in the mushroom bodies was shown to accelerate memory decay after olfactory aversive delay conditioning (Shuai et al., 2010). In trace conditioning, the targeted inhibition of Rac in the mushroom bodies but not in the antennal lobes increased the trace-dependent memory formation. Furthermore, rescue experiments in dopamine (DA) receptor mutants showed that D1 DA receptor expression in mushroom bodies was required for trace conditioning (Shuai et al., 2011).

## Post-odor responses in kenyon cells

KCs have the intriguing property of responding mainly to odor onset, less to odor offset and even less to ongoing odor stimulation (*Schistocerca americana*: Perez-Orive et al., 2002; *Apismellifera*: Szyszka et al., 2005; *Manduca sexta*: Ito et al., 2008; *Drosophila melanogaster*: Murthy et al., 2008; Turner et al., 2008).

In *Manduca sexta*, the probability of KC offset responses increased with stimulus length (Ito et al., 2008), though offset responses were elicited in a different set of KCs than the onset responses (Ito et al., 2008). Reinforcement of the CS offset in behavioral experiments did not result in learning (Ito et al., 2008). But this might be a species or paradigm-specific observation.

KC offset responses in other species have not been examined with respect to their involvement in trace conditioning to date. Indeed, the evidence for an involvement of KCs in trace conditioning (Shuai et al., 2011) and their accepted role in delay conditioning require further investigation of KC post-stimulus activities and their possible trace encoding properties. The trace might be encoded as biochemical tagging of odor-encoding KCs (Wessnitzer et al., 2012). In addition, odor-responsive KCs might become reactivated during the pairing of an odor with a US (Szyszka et al., 2008) and this reactivated KC ensemble might encode the trace (Szyszka et al., 2011).

In vertebrates, delay and trace conditioning rely on different brain structures. Such a distinction on the circuit level might also be true for insects. Exemplified in *Drosophila*, the trace might be encoded by LNs in the antennal lobe (Figure 2), by modulatory neurons like the anterior paired lateral neuron (APL; Liu and Davis, 2009) or the dorsal paired medial neuron (DPM; Waddell et al., 2000), as suggested by Perisse and Waddell (2011) or by other mushroom body extrinsic neurons (Tanaka et al., 2008).

## Molecular requirements of learning during trace and delay conditioning

Behavioral studies revealed not only differences between delay and trace conditioning, but also many similarities, which suggest that the transition between these two forms of learning might be continuous (Menzel, 1983; Tully and Quinn, 1985; Tanimoto et al., 2004; Galili et al., 2011; Szyszka et al., 2011). But do these similarities originate from related molecular mechanisms?

**Figure 3:**
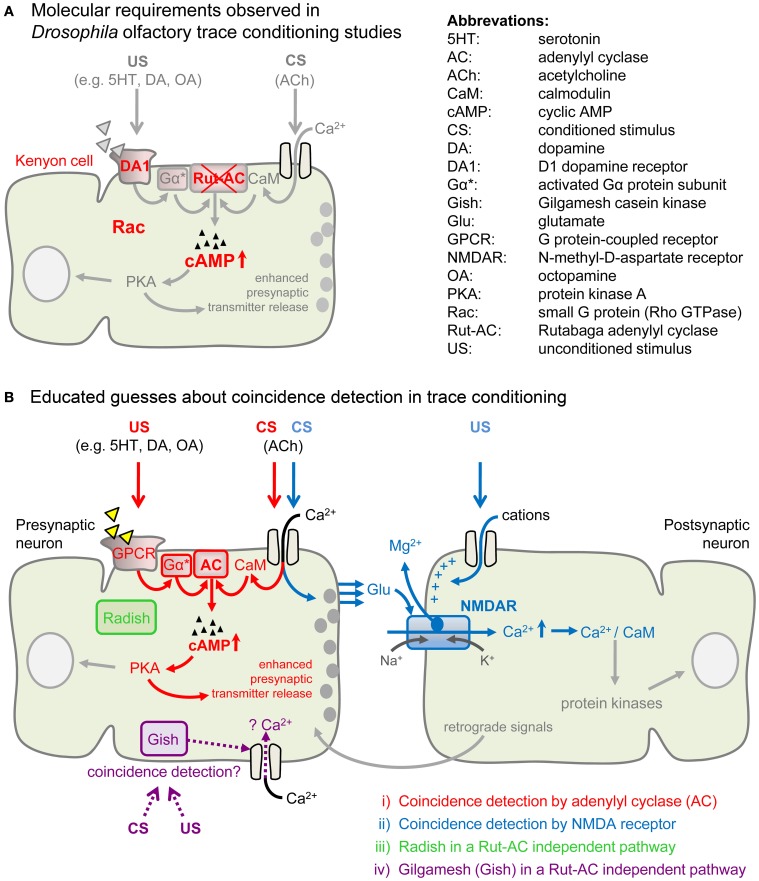
**Molecular requirements for trace conditioning and four non-exclusive models of possible coincidence detection. (A)** Cellular and molecular requirements which were shown to contribute to *Drosophila* olfactory trace conditioning. Shuai et al. (2011) found that the targeted inhibition of Rac in the mushroom bodies increased trace-dependent memory formation. Also D1 dopamine receptor (DA1) expression in mushroom bodies was required for trace conditioning, as shown by rescue experiments (Shuai et al., 2011). Trace conditioning does not require the Rutabaga adenylyl cyclase (Rut-AC; Shuai et al., 2011), but delay and trace conditioning simulations both induced synergistic increases of cAMP (Tomchik and Davis, 2009). **(B)** Educated guesses about coincidence detection in delay conditioning might also apply to trace conditioning. **(i)** Presynaptic coincidence detection by an adenylyl cyclase (AC; shown in red). In the presynaptic neuron, the CS induces Ca^2+^ influx and Ca^2+^ binds to calmodulin (CaM). The US activates G protein-coupled monoaminergic receptors (GPCR) which activate the associated G protein (Gα). When Ca^2+^/CaM complex and activated G protein (Gα^*^) co-occur, the AC is activated more strongly than if they appear alone. This leads to an increased production of cAMP and to activation of protein kinase A (PKA) which enhances presynaptic transmitter release (Heisenberg, 2003). **(ii)** Postsynaptic coincidence detection by the N-methyl-D-aspartate-type glutamate (NMDA) receptor (shown in blue). The CS leads to presynaptic release of glutamate (Glu) which binds to the postsynaptic NMDA receptor. The US, on the other hand, induces the depolarization of the postsynaptic membrane, which allows for the removal of the Mg^2+^ block from the NMDA receptor channel. Opening of the NMDA receptor channel for Ca^2+^ influx is only possible when the CS and the US signal coincide. An elevation of the intracellular Ca^2+^ level leads to the activation of several kinases, inducing synaptic plasticity. The NMDA receptor is involved in delay conditioning in *Drosophila* (Miyashita et al., 2012) and was also shown to be involved in trace conditioning in vertebrates (Gilmartin and Helmstetter, 2010; Czerniawski et al., 2012). A possible role in insect trace conditioning has not yet been investigated. **(iii)** Radish (shown in green) is involved in a Rut-AC independent pathway (Isabel et al., 2004; Folkers et al., 2006) and might contribute to trace conditioning. **(iv)** Gilgamesh (Gish) (shown in purple), a casein kinase I γ homolog in flies, is required for short-term memory formation in *Drosophila* olfactory delay conditioning, functioning independently of Rut-AC and the cAMP pathway (Tan et al., 2010). Hypothetically, Gish mediates increased Ca^2+^ influx upon CS–US coincidence and thus might be a pathway for Rut-AC independent trace conditioning.

Recently, genetic and physiological studies in *Drosophila* gave insights into the molecular requirements of both conditioning forms (Tomchik and Davis, 2009; Shuai et al., 2011). Trace conditioning does not involve the Rutabaga adenylyl cyclase (Rut-AC; Figure 3A; Shuai et al., 2011), which is required for delay conditioning (Duerr and Quinn, 1982; Dudai et al., 1983). Furthermore, the inhibition of Rac enhanced learning performance in *Drosophila* trace conditioning, while delay conditioning remained unaffected (Shuai et al., 2011).

Do these differences derive from the necessity to bridge the temporal gap or from differences in task complexity? In most cases, trace conditioning yields lower memory performance than delay conditioning. Thus weak learning paradigms in general may recruit alternative molecular pathways compared to paradigms which induce strong learning, as suggested in mammal studies (Beylin et al., 2001). In support of this idea, Rac inhibition also enhanced the learning performance in delay conditioning, when it was performed with low odor concentrations, normally leading to low memory performance (Shuai et al., 2011, Supplementals).

Tomchik and Davis (2009) investigated the role of cyclic adenosine monophosphate (cAMP) signaling in delay and trace conditioning. The authors pharmacologically simulated the CS and US by application of acetylcholine (ACh) and DA or octopamine (OA), respectively, onto dissected *Drosophila* brains (Figure 3A). The CS–US timing was chosen according to standard conditioning protocols. The cAMP increase was synergistic for paired ACh–DA applications, compared to the summed response of unpaired applications. This synergistic effect was observed for ACh–DA pairings in both, a delay and a trace conditioning manner (for an ACh–DA interval ≤15 s). The approach revealed no differences between the delay and trace conditioning simulations.

In the delay conditioning simulation the synergistic cAMP increase was Rut-AC dependent, while Rut-AC dependency was not tested in the trace conditioning simulation. According to Shuai et al. (2011) trace conditioning is Rut-AC independent and the observed synergistic cAMP increase (Tomchik and Davis, 2009) thus might be induced by another coincidence detector. Pairing ACh with OA application in a delay conditioning manner resulted in a subadditive effect, which was Rut-AC independent. Simulation of trace conditioning was not tested with OA. These results provide hints for a role of cAMP in trace conditioning (Figure 3A), although it has to be taken into account that the duration of the ACh and DA bath applications is not as precisely controllable as stimuli in behavioral paradigms.

## Searching for the coincidence detector in trace conditioning

As Rut-AC may not be involved in olfactory trace conditioning in *Drosophila* (Shuai et al., 2011), other coincidence detectors may account for the CS–US association. One candidate is the N-methyl-D-aspartate-type glutamate (NMDA) receptor (Traynelis et al., 2010; Miyashita et al., 2012). The Mg^2+^ block of this receptor plays an important role in insect olfactory learning (Miyashita et al., 2012). Only upon correlated activity of a presynaptic and a postsynaptic cell, Mg^2+^ is removed and the channel opens. The resulting large Ca^2+^ influx is crucial for learning (Figure 3B).

From studies in mammals it is known that in some instances NMDA receptors play a role in trace conditioning, but not in delay conditioning. Blocking of NMDA receptor-mediated signaling in the prefrontal cortex of rats modified gene expression pathways in the hippocampus and impaired trace, but not delay fear conditioning (Gilmartin and Helmstetter, 2010; Czerniawski et al., 2012). Could it be that NMDA receptors, in a similar fashion, act as coincidence detectors in insect trace conditioning?

The *radish* gene encodes a protein that is highly expressed in the MBs (Folkers et al., 2006) and was suggested to be involved in Rut-AC independent delay conditioning (Figure 3B; Isabel et al., 2004). Is it possible that Rut-AC independent trace conditioning relies on Radish function as well?

Another candidate for coincidence detection is Gilgamesh (Gish), a casein kinase Iγ homolog. Gish is required for Rut-AC independent olfactory learning in *Drosophila* (Tan et al., 2010) and it accounted for the residual delay learning in Rut-AC and protein kinase A (PKA) mutants (Figure 3B; Skoulakis et al., 1993; Han et al., 2003). Whether and how Gish functions as a coincidence detector is unknown. Gish is supposed to mediate intracellular Ca^2+^ increase in those MB neurons which respond to the reinforced CS (Tan et al., 2010). The study by Tan et al. (2010) showed that delay conditioning is achieved via separate pathways (either Rut-AC or Gish-dependent). Further studies are needed to answer the question of whether the Radish- and/or Gish-dependent pathways are shared in olfactory trace and delay conditioning.

Recent studies in rats have revealed a role for serotonin in mammalian trace conditioning (Miyazaki et al., 2011). The activity of serotonin neurons was increased when rats had to wait for a delayed reward. Serotonin has not yet been tested in insect trace conditioning, and—together with other possible neuromodulators—may be a promising target for future studies.

## Computational models reveal potential mechanisms for trace learning

Computational modeling further supports the intriguing search for the underlying mechanisms of trace conditioning. Several modeling approaches aiming at the neural circuits and/or molecular mechanisms of associative learning might help to understand trace conditioning (Desmond and Moore, 1988; Drew and Abbott, 2006; Izhikevich, 2007; Yarali et al., 2012). The models are based on the mechanism of synaptic plasticity: strengthening the synapses where stimuli coincide.

A process accounting for association on millisecond timescale is spike timing dependent plasticity (STDP) which is involved in both long term potentiation and long term depression of synapses. A synapse is strengthened and synaptic transmission is increased when presynaptic action potential firing precedes postsynaptic firing within a short time window of a few milliseconds. The reverse order weakens the synapse and reduces synaptic transmission.

In associative conditioning, pre- and postsynaptic firing induced by CS and US, respectively, would result in synaptic strengthening. When the CS is presented alone after many pre–post pairings, the post-neuron might fire without a US input. This strengthened synaptic connection reflects associative learning.

However, there is a timescale discrepancy regarding stimulus timing in behavior and STDP (reviewed in: Gallistel and Matzel, 2013). On the behavioral level, actions often elapse over several seconds, while the physiological timescale of STDP extends only over milliseconds. In delay conditioning the CS spikes could overlap with the US spikes and thus lead to potentiation of those synapses. In trace conditioning, this coincidence would not be possible since the CS and the US are several seconds apart.

To account for this discrepancy, Drew and Abbott (2006) assumed in their model that a CS evokes in the presynaptic neurons long spike trains of action potentials with slowly decaying spike rates after stimulus offset. The residual spiking serves as a trace and can coincide with the postsynaptic US spiking, increasing the synaptic strength. In this model, repeated pairing of CS–US led to potentiation of the synaptic efficacy, enabling postsynaptic firing from presynaptic activation alone. The incorporation of slow firing rate decays into the STDP model solved the observed timing problem for trace conditioning. However, the key assumption of this model (that long spike trains follow stimulus termination) contradicts with the physiological findings in olfactory learning. The KCs, which are assumed to be the site of CS–US coincidence, do not evoke such long spike trains, but only very sparse and short-lasting responses upon odor application (Szyszka et al., 2005; Ito et al., 2008).

**Figure 4:**
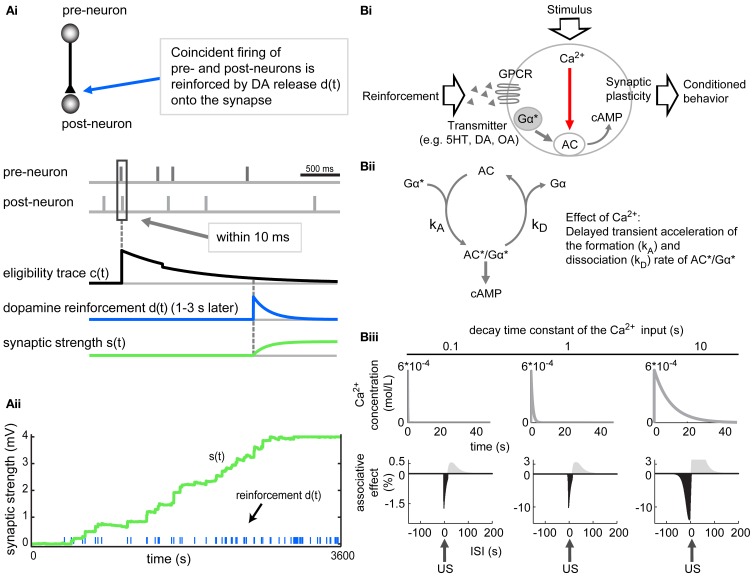
**Models relying on spike timing dependent plasticity (STDP) or biochemical processes can account for trace processes. (Ai)** In the model by Izhikevich (2007), the coincident firing of a pre- and then a postsynaptic neuron (within 10 ms; marked by a rectangle) elicits a synaptic eligibility trace c(t) in the corresponding synapse. This eligibility trace decays exponentially to zero. Reinforcement d(t), here a dopamine (DA) release delayed by 1–3 s in combination with the residual eligibility trace, increases the synaptic strength s(t) [s(t) = c × d] of the particular synapse. **(Aii)** Repeated reinforcement of such a pre–post firing event increasingly strengthens the particular synapse. This in turn increases the probability of coincident firings of this synapse. Adapted from Izhikevich (2007), with permission. **(B)** Lingering Ca^2+^ and coincidence detection by an adenylyl cyclase (AC) might account for trace conditioning in the model by Yarali et al. (2012). **(Bi)** Ca^2+^ influx and Gα activation (induced by CS and US, respectively) synergistically act on the AC, leading to increased cAMP production and strengthening of the synaptic output. **(Bii)** In this model Ca^2+^ is supposed to transiently accelerate both the formation and dissociation rates (k_A_ and k_D_) of the AC^*^/Gα^*^ complex to the same extent. When the system is in equilibrium (k_A_ and k_D_ are the same), Ca^2+^ has no effect on the cAMP level. But when Ca^2+^ influx shortly precedes the transmitter induced activation of Gα^*^, the formation (k_A_) of AC^*^/Gα^*^ is at this time point the dominant reaction. This leads to a rise in AC^*^/Gα^*^ concentration and thus, enhanced cAMP production. When Ca^2+^ influx follows Gα^*^, the dissociation of AC^*^/Gα^*^ is promoted, leading to decreased cAMP production. **(Biii)** This model can account for trace conditioning by changing the Ca^2+^ decay time constants (different decay time constants chosen are 0.1, 1 and 10 s). The larger decay constants (e.g., 10 s) cause a long tail of Ca^2+^ transient (upper row). This allows for associations of stimuli over longer interstimulus intervals (ISIs; bottom row) and is critical for reproducing the behavioral measurements of trace conditioning. The longer the Ca^2+^ decay time is, the larger the negative “associative” effect is in the simulation. This reveals that lingering Ca^2+^ in KCs might contribute to bridge the temporal gap between CS and US. Note that in this model the US onset is set to 0 and the CS onset shifts to the left for increasing ISIs (CS–US intervals). The negative associative effects correspond to the learned odor avoidance in olfactory aversive delay and trace conditioning. The Ca^2+^ influx is always constant (rising to a Ca^2+^ concentration peak of 6 × 10^-4^ mol/L within 40 ms). Adapted from Yarali et al. (2012).

Other models suggested that the combination of STDP and neuromodulators might solve the timescale discrepancy and explain coincidence detection in trace conditioning. Izhikevich (2007) suggested a network where transient synaptic changes, induced by coincident pre- and postsynaptic spiking (following the STDP rule), were enhanced by a DA reinforcement (Figure 4Ai). These transient synaptic changes—acting as synaptic eligibility traces—could be the activation of an enzyme with slow kinetics, important for synaptic plasticity. In the model, these eligibility traces were exponentially decaying over several seconds. During this decay, the synapse got reinforced by a global DA release (1–3 s after the STDP; Figure 4Ai) leading to a reinforcement of the synaptic eligibility trace and strengthening of the synapse. Other synapses in the network that also elicited coincident firing which was not linked to the reward, were not strengthened. Repetition of reinforcing each such pre–post firing event increasingly strengthened the particular synapse. This in turn increased the probability of coincident firings at this synapse, leading to even more reinforcement (Figure 4Aii). The model shows how STDP might also contribute to insect trace learning when the fast STDP mechanism is combined with slower biochemical processes and subsequently mediated by neuromodulators.

This idea was experimentally tested in the mushroom bodies of locusts. Cassenaer and Laurent (2012) examined the effect of neuromodulators (specifically OA) on the plasticity of KC output synapses onto their postsynaptic targets, the beta-lobe neurons. The synapses at which pre- and postsynaptic action potentials were coinciding seemed to be tagged, and only the tagged synapses were subsequently modified by OA, which was applied 1 s after the STDP. This process could underlie delay and also trace conditioning as the temporal gap in trace conditioning might be bridged by the synaptic eligibility trace (Izhikevich, 2007), and specific synapses would then be reinforced by the neuromodulator. With respect to trace conditioning, it would be interesting to know if gaps longer than 1 s between the STDP and the application of neuromodulators have an effect on synaptic plasticity, and whether gap length and corresponding synaptic plasticity fit to behavioral observations.

In addition to STDP, other mechanisms have been proposed to account for associative learning. The model by Yarali et al. (2012) refers to aversive olfactory learning in *Drosophila melanogaster* and is based on the mechanism of coincidence detection by an adenylyl cyclase (AC). It suggests that slowly decaying Ca^2+^ transient in the presynaptic neuron, elicited by the CS, could function as a stimulus trace. The odor-induced Ca^2+^ signal (Wang et al., 2004; Yu et al., 2006; Wang et al., 2008; Honegger et al., 2011) and the shock-induced DA signal (Schwaerzel et al., 2003; Riemensperger et al., 2005; Kim et al., 2007; Claridge-Chang et al., 2009; Aso et al., 2010) converge in the mushroom body KCs, where they synergistically activate an AC (Figure 4Bi). The activation of the AC by the US signal (via an activated G protein subunit, Gα^*^) is bidirectionally modulated by the CS-induced Ca^2+^ influx depending on the relative timing of the CS and the US (Figure 4Bii). The Ca^2+^ influx transiently increases the rate constants for both the formation and the dissociation (k_A_ and k_D_, Figure 4Bii) of the active AC^*^/Gα^*^ complex.

Based on this mechanism of coincidence detection by the AC, odor-shock conditioning in *Drosophila* was simulated. When the odor-induced Ca^2+^ influx shortly preceded the US-induced G protein activation (Gα^*^) as in delay conditioning, the formation of the AC^*^/Gα^*^ complex was transiently accelerated. This led to increased cAMP production resulting in potentiation of synaptic output in these particular KCs. In trace conditioning where the CS is already gone upon US arrival, the coincidence could be achieved by residual Ca^2+^ transient in the cell.

To test if the model is capable of predicting trace conditioning, the authors changed the shape of the Ca^2+^ signal such that at the moment of US arrival, there was still sufficient Ca^2+^ present to induce plasticity (Figure 4Biii). This residual Ca^2+^ was critical for reproducing the behavioral measurements of trace conditioning. The slower the simulated decay of Ca^2+^ was the larger was the “associative” effect in the simulation (Figure 4Biii). Thus lingering Ca^2+^ in KCs could contribute to bridge the temporal gap between two stimuli. In *in vivo* studies long-lasting Ca^2+^ concentration in KCs was neither confirmed nor excluded (Wang et al., 2004; Yu et al., 2006; Wang et al., 2008).

This model (Yarali et al., 2012) gives a simple biochemical explanation for delay and trace conditioning based on the modulation of AC activation by the transient Ca^2+^ level. The components of this model, namely the cAMP formation by the AC have been experimentally investigated by Tomchik and Davis (2009). Synergistic increase of cAMP in α and α′ lobes of the mushroom bodies was induced by pharmacologically mimicking CS and US in dissected *Drosophila* brains. Moreover, the cAMP pathway itself was shown to be strongly involved in learning (Gervasi et al., 2010).

Note that some of the described models cannot account for 1-trial trace conditioning since they are based on repeated stimulus pairings.

## Methodological considerations

The variety of trace conditioning paradigms renders a comparison of the obtained results rather difficult. Each method has its own peculiarity, such as the properties of the chosen CS or US. According to the Rescorla and Wagner model for classical conditioning (Rescorla and Wagner, 1972), learning directly depends on the salience and intensity of the CS and the US. Given that trace conditioning in most cases is less efficient than delay conditioning, this difference can be explained by a reduced CS salience in trace conditioning. The CS salience probably decays until the US is applied. Not only does the length of the CS–US interval have considerable impact on the CS salience, but so does the CS identity (Pavlov, 1927). Thus, trace conditioning studies using different CS are not necessarily comparable.

Some CS modalities hold potential pitfalls, as shown for the very common olfactory trace conditioning paradigms. We found that many odors are “sticky” and linger in the training device (Galili et al., 2011), such that it is impossible to clearly distinguish between trace and delay conditioning. Therefore, proper controls are important to exclude residual odor in the training device, e.g., behavioral controls such as unpaired stimulus presentation (Galili et al., 2011), physiological controls such as calcium imaging from olfactory neurons (Szyszka et al., 2011) or technical controls such as photoionization measurements (Shuai et al., 2011).

What other kinds of stimulus modalities seem suitable for trace conditioning? There are several studies indicating that visual stimuli are promising. To our knowledge the first report about visual trace conditioning in insects is from the early 1930s. Opfinger (1931) demonstrated that the color presented during the approach of a food source is learned better by honeybees than the color presented during feeding. *Drosophila* are also able to remember visual stimuli. They can remember the position of a vanished visual object and use this information for navigation (Neuser et al., 2008). In the past 50 years, several visual trace conditioning studies have been carried out (Menzel, 1968; Grossmann, 1970, 1971; Menzel and Bitterman, 1983) showing that visual stimuli are well suited to study this learning form.

The sensory pathways underlying trace conditioning certainly depend on the stimulus modality. However, the shape of the CS–US interval function in visual and olfactory conditioning looks very similar (Menzel and Bitterman, 1983). Thus the cellular mechanisms for keeping the CS trace may be related in different modalities. It is also evident that the suitability of the US for conditioning paradigms depends on the responsiveness of the animal (Pavlov, 1927).

## Conclusions

In this review we described recent findings regarding the behavioral, molecular, physiological, and modeling aspects of insect trace conditioning. We noted some differences in the features of trace conditioning between different studies. For instance, in bees the initial part of a stimulus initiates the stimulus trace whereas it seems to be the end of a stimulus that initiates the trace in *Drosophila*. In bees and *Drosophila*, trace conditioning seems to yield lower memory performance than delay conditioning paradigms, whereas it is the opposite in *Manduca sexta*. Whether these are species-specific differences caused by adaptation to diverse natural habitats or paradigm-dependent differences remains to be shown. Comparing trace conditioning between similar paradigms in different species and between different paradigms in the same species might give the answer. We also highlighted many common properties of trace conditioning. One example is the commonly shared shape of CS–US interval function across species and paradigms. Such communalities make us believe that an integrative approach will be auspicious for revealing the fundamental mechanisms behind trace conditioning. Insects are perfectly suited for such a comparison because they learn quickly, and they allow for a rich repertoire of conditioning paradigms. These include, among others, appetitive olfactory conditioning in honeybees (Matsumoto et al., 2012; Menzel, 2012), bumble bees (Riveros and Gronenberg, 2009), *Drosophila* (Tempel et al., 1983; Chabaud et al., 2006), ants (Guerrieri and d'Ettorre, 2010), *Manduca sexta* (Ito et al., 2008) and locusts (Simoes et al., 2011), aversive olfactory conditioning in *Drosophila* (Tully and Quinn, 1985) and honeybees (Abramson, 1986; Vergoz et al., 2007), visual conditioning in honeybees (Dobrin and Fahrbach, 2012), and auditory conditioning in *Drosophila* (Menda et al., 2011).

Salience of a CS and US influence the strength of associative memories (Rescorla and Wagner, 1972). Compared to delay conditioning, the generally lower performance in trace conditioning could reflect a lower salience of the CS and/or US. It will therefore be interesting to study how the salience of both the CS and the US influences learning and memory in trace conditioning. Could one reach the same stimulus salience and thus equal acquisition and memory performance in trace and delay conditioning?

In this review, we discussed alternative mechanisms that may account for trace conditioning, such as recurrent neuronal firing, residual Ca^2+^ transients, slowly decaying eligibility traces and different coincidence detectors apart from the well-studied Rut-AC. We are still far away from understanding how stimulus traces are encoded in the brain and how the coincidence detection between a stimulus trace and the US is achieved. Do trace and delay conditioning in insects engage different neural circuits, as is the case in vertebrates? *Drosophila*, with the possibility to genetically manipulate identifiable neurons, appears to us as the most promising model, as it allows a truly integrative approach to address these questions from molecular to circuit level.

### Conflict of interest statement

The authors declare that the research was conducted in the absence of any commercial or financial relationships that could be construed as a potential conflict of interest.
